# The Moving Rubber Hand Illusion Reveals that Explicit Sense of Agency for Tapping Movements Is Preserved in Functional Movement Disorders

**DOI:** 10.3389/fnhum.2017.00291

**Published:** 2017-06-06

**Authors:** Angela Marotta, Federica Bombieri, Massimiliano Zampini, Federico Schena, Carlo Dallocchio, Mirta Fiorio, Michele Tinazzi

**Affiliations:** ^1^Department of Neurosciences, Biomedicine and Movement Sciences, University of VeronaVerona, Italy; ^2^Neurology Unit, Neuroscience Department, Azienda Ospedaliera Universitaria IntegrataVerona, Italy; ^3^CiMeC Center for Mind/Brain Sciences, University of TrentoRovereto, Italy; ^4^Department of Psychology and Cognitive Science, University of TrentoRovereto, Italy; ^5^Division of Neurology, Civil Hospital, Azienda Ospedaliera della Provincia di PaviaVoghera, Italy

**Keywords:** self-recognition, bodily actions, sense of agency, sense of body ownership, functional movement disorders, rubber hand illusion

## Abstract

Functional movement disorders (FMD) are characterized by motor symptoms (e.g., tremor, gait disorder, and dystonia) that are not compatible with movement abnormalities related to a known organic cause. One key clinical feature of FMD is that motor symptoms are similar to voluntary movements but are subjectively experienced as involuntary by patients. This gap might be related to abnormal self-recognition of bodily action, which involves two main components: sense of agency and sense of body ownership. The aim of this study was to systematically investigate whether this function is altered in FMD, specifically focusing on the subjective feeling of agency, body ownership, and their interaction during normal voluntary movements. Patients with FMD (*n* = 21) and healthy controls (*n* = 21) underwent the moving Rubber Hand Illusion (mRHI), in which passive and active movements can differentially elicit agency, ownership or both. Explicit measures of agency and ownership were obtained via a questionnaire. Patients and controls showed a similar pattern of response: when the rubber hand was in a plausible posture, active movements elicited strong agency and ownership; implausible posture of the rubber hand abolished ownership but not agency; passive movements suppressed agency but not ownership. These findings suggest that explicit sense of agency and body ownership are preserved in FMD. The latter finding is shared by a previous study in FMD using a static version of the RHI, whereas the former appears to contrast with studies demonstrating altered implicit measures of agency (e.g., sensory attenuation). Our study extends previous findings by suggesting that in FMD: (i) the sense of body ownership is retained also when interacting with the motor system; (ii) the subjective experience of agency for voluntary tapping movements, as measured by means of mRHI, is preserved.

## Introduction

The experience of the body as one’s own (i.e., sense of body ownership) and the sense of authorship over movements (i.e., sense of agency) are basic cognitive components of self-recognition of bodily actions (van den Bos and Jeannerod, 2002; Jeannerod, 2003; Tsakiris et al., 2007; Louzolo et al., 2015). This cognitive function is taken for granted in everyday life, although it appears to be altered in some pathological conditions, like functional movement disorders (FMD).

Functional movement disorders are characterized by the presence of motor symptoms in the absence of neurological disease (Edwards et al., 2013). The most common form is tremor, followed by dystonia and myoclonus (Factor et al., 1995; Bhatia and Shneider, 2007; Edwards and Bhatia, 2012). Pure functional gait disorder accounts for about 6% of patients, although abnormal gait is commonly present in association with other forms of FMD (Baik and Lang, 2007; Edwards and Bhatia, 2012). Regardless of the phenomenology, there are common clinical features characterizing FMD, such as sudden onset of symptoms, rapid progression, variability in symptom severity, and past history of other functional motor symptoms (Edwards and Bhatia, 2012). However, one key feature of FMD that distinguishes it from organic movement disorders is that the motor symptoms resemble voluntary movements (e.g., functional tremor disappears with distraction) but are perceived as involuntary by the patient (Hallett, 2010, 2016; Edwards et al., 2011). In other words, although the motor symptoms look like intentionally produced movements, the patients’ self-report is that the abnormal movements are not under their control (Edwards et al., 2013).

Because of their similarity with voluntary movements, functional motor symptoms are often interpreted as being feigned by patients (Macerollo et al., 2015). Recent evidence against this interpretation suggests, however, that the dissociation between the voluntary nature of motor symptoms and the sense of involuntariness reported by patients could hint at a lack of sense of agency (Voon et al., 2010, 2011; Edwards et al., 2011; Kranick et al., 2013; Pareés et al., 2014; Macerollo et al., 2015).

The sense of agency is the feeling of control over one’s own action. This basic cognitive function allows to distinguish between self-generated and externally generated movements (David et al., 2016). According to computational models of motor control such as the comparator model (Frith et al., 2000; Blakemore et al., 2002; Chambon et al., 2014), a sense of agency arises from the matching between the predicted and the actual sensory outcome of intended actions. In this view, a voluntary action starts with the intention to achieve a desired state, with a motor command generated to reach the goal as a consequence. Additionally, a predictive component within the motor system uses a copy of the motor command (so-called efference copy) to predict the sensory outcome of the intended movement. This prediction is then compared to the actual sensory outcome, and in case they match, the sense of agency arises (Frith et al., 2000; Blakemore et al., 2002; Chambon et al., 2014; Haggard, 2017). A sensory consequence of the comparison between the predicted and the actual sensory outcome is the so-called sensory attenuation.

Sensory attenuation refers to perceiving a sensory event as less intense when it occurs in association with a voluntary, but not with an involuntary, movement (Blakemore et al., 1998, 2000). A loss of sensory attenuation during self-generated movements (e.g., abduction of the thumb) has been found in different types of FMD as compared to healthy volunteers (Pareés et al., 2014; Macerollo et al., 2015). This finding was interpreted as behavioral evidence for a reduced sense of agency in FMD.

In the same line, another study applied a different behavioral paradigm, such as intentional binding, as an implicit measure of agency in FMD (Kranick et al., 2013). Intentional binding refers to the subjective temporal attraction between a voluntary action and its sensory outcome (Haggard et al., 2002). When an action (e.g., pressing a button) is executed voluntarily, participants perceive the action and its sensory outcome as temporally closer together than they actually are. This effect does not occur for involuntary movements, however (Haggard et al., 2002). Intentional binding is decreased in FMD patients as compared to healthy controls, further supporting the hypothesis of an altered sense of agency in FMD (Kranick et al., 2013).

All these studies have been important in demonstrating that the sense of agency in FMD is altered according to implicit measures. Of note, however, the tasks described above required participants to judge the time of performed movement or its perceptual consequences, rather than the feeling of control over bodily movement. Hence, no study to date has linked the sense of agency to the perception of bodily movements in FMD.

Here, we investigated whether patients with FMD can recognize an observed bodily movement as self-produced or externally generated. To this aim, we applied the moving rubber hand illusion (mRHI) paradigm, in which participants experience a sense of agency by observing a rubber hand actively moved by their own hidden hand (Kalckert and Ehrsson, 2012). In addition, the mRHI allows to investigate the sense of body ownership, the other main component of self-recognition of bodily actions (van den Bos and Jeannerod, 2002; Jeannerod, 2003; Tsakiris et al., 2007; Louzolo et al., 2015).

While the sense of agency is the feeling of control over bodily moments, the sense of body ownership is the feeling that the moving body part belongs to the self. The sense of body ownership arises from the multisensory integration of different sensory signals (e.g., visual, tactile, and proprioceptive) coming from the body. More precisely, when the brain receives temporally and spatially congruent sensory input from a body part, that body part is experienced as belonging to oneself (Tsakiris et al., 2007). During voluntary movements, the sense of agency and the sense of body ownership coincide. In case of involuntary movements, instead, it is possible to distinguish one from the other. That is, a moving body part is still perceived as belonging to the self even though an external agent causes the movement. This suggests that the sense of body ownership can be consistent with a lack of sense of agency (Gallagher, 2000). The sense of body ownership and the sense of agency, although independent, may strongly influence each other (Newport et al., 2010; Tsakiris et al., 2010).

In the mRHI paradigm, by manipulating the agent of the movement (self or other) and the posture of the rubber hand (plausible or implausible), we can investigate the sense of agency and the sense of body ownership separately or in interaction. For instance, when the movement is passively induced by the experimenter, the sense of body ownership over the visible hand is retained, whereas the sense of agency is abolished. Conversely, active movements of a rotated rubber hand induce an opposite pattern, with the sense of agency retained and the sense of body ownership abolished (Kalckert and Ehrsson, 2012).

That movements interact with the sense of the body can be inferred from previous studies that applied the classical RHI paradigm in pathological populations (Fiorio et al., 2011; Lenggenhager et al., 2012; Scandola et al., 2014; Tidoni et al., 2014; Burin et al., 2015). More precisely, patients with impaired movement execution due to various different causes, including dystonia (Fiorio et al., 2011), spinal cord injury (Lenggenhager et al., 2012; Scandola et al., 2014; Tidoni et al., 2014) or stroke (Burin et al., 2015), present with a weaker sense of body ownership. Furthermore, a recent TMS study in healthy participants showed that excitability of the primary motor cortex interacts with body ownership (Della Gatta et al., 2016). The mRHI paradigm allows to directly investigate the link between movements and the sense of the body. Hence, by applying the mRHI paradigm in FMD patients and healthy controls, we expected to find a specific pattern of results in the different experimental conditions. In the condition that selectively induces a sense of body ownership (but not agency), we predicted similar performance in patients and controls, as demonstrated in a recent study with a static version of the RHI paradigm (Demartini et al., 2016). Instead, in the condition that selectively induces a sense of agency (but not body ownership), we would expect an alteration in FMD, which resembles a lack of control over motor symptoms. Finally, according to previous findings demonstrating that a lack of agency may prevent ownership (Newport et al., 2010), we would expect a reduced sense of ownership related to the altered sense of agency in the condition eliciting both agency and ownership.

## Materials and Methods

### Participants

Twenty-one patients [19 women; mean age ± standard deviation (SD), 39.48 ± 12.84 years] with clinically defined diagnosis of FMD were recruited from the Neurology Section, University Hospital of Verona, and the Neurology Division, Voghera Civil Hospital (see **Table 1** for clinical and demographic characteristics). Diagnostic criteria for FMD patients referred to the latest version of the Diagnostic and Statistical Manual of Mental Disorders, Fifth edition (DSM-5) (American Psychiatric Association, 2013).

**Table 1:** Demographic and clinical data for patients.

Patient/gender	Age	Disease duration (months)	Type and localization of functional symptoms	Medication
1/F	36	1	Gait disorder	Amitriptyline
2/F	44	84	Gait disorder	Clonazepam
3/F	66	84	Gait disorder	None
4/F	44	10	Gait disorder	Escitalopram Alprazolam
5/F	37	8	Gait disorder, Dystonia (neck)	Delorazepam
6/F	40	18	Gait disorder, Dystonia (both legs)	Amitriptyline
7/F	44	24	Gait disorder	None
8/F	34	n.a.	Gait disorder	None
9/F	36	6	Gait disorder, Tremor (both hands)	Citalopram
10/F	26	40	Gait disorder	Venlafaxine
11/F	32	32	Gait disorder	None
12/M	52	3	Gait disorder, Tremor (both hands)	Duloxetine Diazepam
13/F	38	36	Gait disorder	Bromazepam
14/F	29	36	Gait disorder, Tremor (hands and legs)	Duloxetine Lorazepam
15/F	53	48	Gait disorder, Tremor (right hand)	Escitalopram
16/F	23	3	Gait disorder, Dystonia (neck), Tremor (both hands)	Amitriptyline Diazepam
17/F	30	6	Tremor (both hands)	Amitriptyline Delorazepam
18/M	30	2	Tremor (right hand)	None
19/F	22	10	Gait disorder, Dystonia (neck), Tremor (both hands)	None
20/F	70	12	Tremor (both hands)	Alprazolam
21/F	51	36	Tremor (left hand)	None

*n.a., not available.*

Exclusion criteria were sensory abnormalities and other major neurological disorders. Twenty-one healthy volunteers (17 women; mean age ± SD, 45.24 ± 11.86 years) were recruited for the control group at the Department of Neurosciences, Biomedicine and Movement Sciences, University of Verona. All participants were right-handed. The study was approved by the ethical committee of the University Hospital of Verona. Participants gave their written informed consent prior to participation. The experiment was carried out in accordance with the principles of the Declaration of Helsinki.

### mRHI Set-Up

**FIGURE 1:**
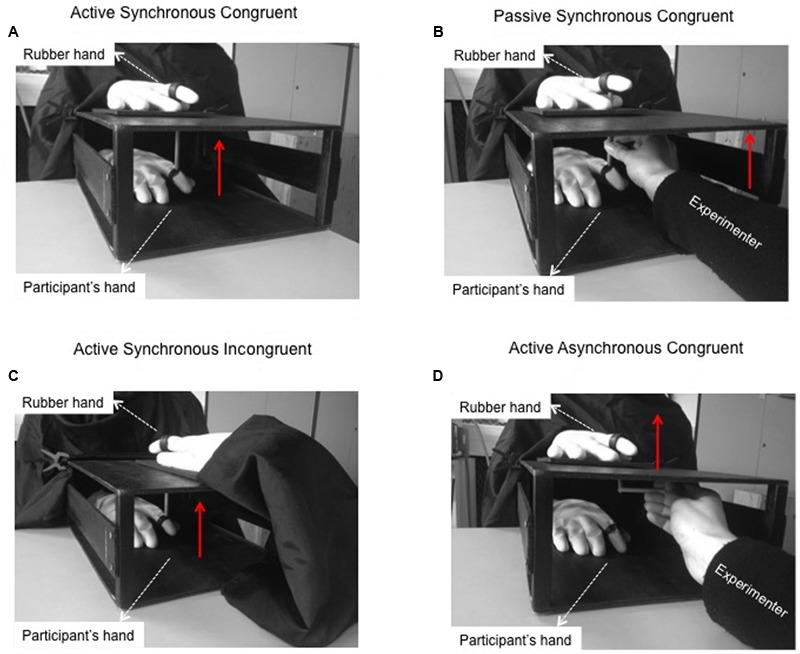
Moving Rubber Hand Illusion apparatus. **(A)** In the Active Synchronous Congruent condition, when the participant actively moved her own index finger, the rubber hand moved in turn. This condition usually evokes the sense of agency *and* the sense of ownership over the rubber hand. The red arrow indicates the direction of the movement. **(B)** In the Passive Synchronous Congruent condition, the experimenter moved a wooden stick, thus causing both the participant’s hand and the rubber hand to move. This condition usually induces ownership but *not* agency over the rubber hand. **(C)** In the Active Synchronous Incongruent condition, the rubber hand was rotated of 180 degrees with respect to the participant’s hand. The participant moved her index finger and the rubber hand moved synchronously. This condition usually evokes agency but *not* ownership over the rubber hand. **(D)** In the Active Asynchronous Congruent condition, the rubber hand was placed in an anatomically congruent position and the participant performed active movements as in the Active Synchronous Congruent condition. In this case, however, the rubber hand was moved by the experimenter with a short delay with respect to the onset of the participant’s movement. This condition does not evoke sense of agency or sense of body ownership.

Participants sat at a table with their right hand hidden from view inside a box (35 cm × 25 cm × 12 cm). A realistic artificial right hand fashioned from a yellow, cotton-filled rubber glove (distance from the wrist to the index fingertip, 25 cm) was positioned on top of the box (**Figure 1A**). Before starting the experiment, participants donned a yellow glove to make the real hand appear as similar as possible to the rubber hand. The index fingertips of the participant’s hand and the rubber hand were inserted into plastic rings that were connected to a wooden stick (**Figure 1A**). A black drape connected to the box covered the participant’s right arm and shoulder.

### Procedure

The experiment was carried out in a single session lasting about 1 h (comprising all the phases of the procedure, from consent form collection to the end of the real experiment). The task entailed watching the rubber hand while performing tapping movements with the right index finger. The finger movements were performed following a semi-regular rhythm, in which double taps were executed at random intervals. More precisely, the participants had to perform tapping movements with the right index finger on a table, following a rhythm of about 1 Hz (as described in Kalckert and Ehrsson, 2012). In order to avoid a regular rhythm of the movement, which could have prevented the illusion (Kalckert and Ehrsson, 2012), we told the participants they could vary the duration of the breaks between two consecutive taps at their own discretion. Prior to the experiment, the participants were taught the semi-regular tapping rhythm with the aid of a metronome set at 1 Hz (not present during the experiment). In order to be sure that the participants had correctly learned and retained the acquired semi-regular rhythm, the last part of the training was performed without the help of the metronome (as in the real experiment). The experimenter showed the participants how to perform the movements and corrected any mistakes in executing the movements (e.g., regular instead of irregular pattern, single instead of double tapping, etc.).

We applied four experimental conditions which differed for three parameters: type of movement (active vs. passive), relative position of the rubber hand and the participant’s hand (congruent vs. incongruent), synchrony between the movement of the participant’s hand and the rubber hand (synchronous vs. asynchronous). A detailed description of the four experimental conditions is given below.

#### Active Synchronous Congruent Condition (**Figure 1A**)

The participant’s index finger was connected with a wooden stick to the index finger of the rubber hand. When the participant actively moved her hidden index finger, the index finger of the visible rubber hand moved in synchrony. This condition usually evokes a strong illusion of ownership over the rubber hand, as well as a strong sense of agency on the observed movement (Kalckert and Ehrsson, 2012).

#### Passive Synchronous Congruent Condition (**Figure 1B**)

The index fingers of the participant’s hand and the rubber hand were connected with a wooden stick. The participant was instructed to relax her hand while the experimenter moved the two index fingers using the wooden stick. This condition usually evokes a strong illusion of ownership over the rubber hand but not a feeling of agency, since the participant does not actively control the movement of the rubber hand (Kalckert and Ehrsson, 2012).

#### Active Synchronous Incongruent Condition (**Figure 1C**)

The rubber hand was rotated 180 degrees with respect to the position of the participant’s hand. The two hands were connected with a wooden stick and the participant was asked to actively move her hidden index finger and to watch the visible rubber finger moving in synchrony. In this condition, the participant usually feels agency but not ownership because of the incongruent posture of the rubber hand.

#### Active Asynchronous Congruent Condition (**Figure 1D**)

The participant’s hand and the rubber hand were disconnected from one another. Concealed behind a panel, the experimenter moved the finger of the rubber hand immediately (∼500 ms) after the participant moved her finger. Although in this condition the rubber hand is in a plausible posture, the asynchrony between the two movements disrupts both ownership and agency.

Each trial lasted 90 s and was performed only once, resulting in a total of four experimental trials.

### mRHI Questionnaire

To measure the sense of ownership and sense of agency, we used the Italian version of the 16-statement questionnaire devised by Kalckert and Ehrsson (2012) (**Table 2**). The questionnaire statements can be grouped into four categories: four statements concern the illusory sense of ownership (e.g., “*I felt as if the rubber hand was my hand*”) (ownership-statements), four statements are related to sense of agency (e.g., “*I felt as if I caused the movement I saw*”) (agency-statements), and eight statements serve as controls, four for the sense of ownership (ownership-control) and four for the sense of agency (agency-control) (Kalckert and Ehrsson, 2012, 2014; Jenkinson and Preston, 2015). The control statements are not related to the subjective experience of ownership and agency, but serve to rule out confounding factors like compliance, suggestibility, and expectancy effects (e.g., ownership-control: “*It seems as if I had more than one right hand*”, agency-control: “*I felt as if the rubber hand was controlling my will*”) (Kalckert and Ehrsson, 2012, 2014; Jenkinson and Preston, 2015).

**Table 2:** The moving Rubber Hand Illusion Questionnaire.

English version (by Kalckert and Ehrsson, 2012)	Italian version
**Ownership-statements**	
(1) I felt as if I was looking at my own hand.	(1) Mi sentivo come se stessi guardando la mia mano.
(2) I felt as if the rubber hand was part of my body.	(2) Mi sentivo come se la mano di gomma fosse parte del mio corpo.
(3) It seemed as if I were sensing the movement of my finger in the location where the rubber finger moved.	(3) Sembrava come se sentissi il movimento del mio dito nello stesso punto in cui si muoveva il dito della mano di gomma.
(4) I felt as if the rubber hand was my hand.	(4) Mi sentivo come se la mano di gomma fosse la mia mano.
**Ownership-control**	
(5) I felt as if my real hand were turning rubbery.	(5) Mi sentivo come se la mia mano stesse diventando di gomma.
(6) It seems as if I had more than one right hand.	(6) Sembrava come se io avessi più di una mano destra/sinistra.
(7) It appeared as if the rubber hand were drifting toward my real hand.	(7) Sembrava come se la mano di gomma si spostasse verso la mia mano.
(8) It felt as if I had no longer a right hand, as if my right hand had disappeared.	(8) Mi sentivo come se non avessi più la mano destra, come se la mia mano destra fosse scomparsa.
**Agency-statements**	
(9) The rubber hand moved just like I wanted it to, as if it was obeying my will.	(9) La mano di gomma si muoveva proprio come volevo, come se stesse obbedendo alla mia volontà.
(10) I felt as if I was controlling the movements of the rubber hand.	(10) Mi sentivo come se stessi controllando i movimenti della mano di gomma.
(11) I felt as if I was causing the movement I saw.	(11) Mi sentivo come se io stessi causando i movimenti che vedevo.
(12) Whenever I moved my finger I expected the rubber finger to move in the same way.	(12) Ogni volta che muovevo il mio dito mi aspettavo che il dito di gomma si muovesse nello stesso modo.
**Agency-control**	
(13) I felt as if the rubber hand was controlling my will.	(13) Mi sentivo come se la mano di gomma stesse controllando la mia volontà.
(14) I felt as if the rubber hand was controlling my movements.	(14) Mi sentivo come se la mano di gomma stesse controllando i miei movimenti.
(15) I could sense the movement from somewhere between my real hand and the rubber hand.	(15) Mi è sembrato di percepire il movimento da qualche parte tra la mia mano e la mano di gomma.
(16) It seemed as if the rubber hand had a will of its own.	(16) Sembrava come se la mano di gomma avesse una volontà propria.

At the end of each trial, the 16 statements were presented in random order and the participant was asked to rate her agreement on a 7-point Likert scale from -3 (“totally disagree”) to +3 (“totally agree”), with 0 (“uncertain”) indicating neither agreement nor disagreement (Kalckert and Ehrsson, 2012).

### Data Analyses

Demographic data were analyzed by means of a *t-*test (age) and a chi-squared test (gender) to verify whether the two groups were comparable for age and gender distribution. The ratings given to four statements in each category were averaged together to obtain a mean score for ownership-statements, agency-statements, ownership-controls, and agency-controls. Data were first assessed for normal distribution with the Shapiro–Wilk test. Since the data were not normally distributed (*p* < 0.05), non-parametric tests were applied. The statistical design was planned stepwise as follows:

First, the Wilcoxon signed-rank test was used to compare the mean value of the ownership-statements and agency-statements with the mean value of the respective ownership-control and agency-control, in each experimental condition and in each group separately. These comparisons allowed to check the reliability of the mRHI paradigm in inducing a sense of body ownership and a sense of agency. In order to test whether the scores at the experimental statements (for both ownership and agency) were significantly different from zero (that indicates “uncertainty”) in specific conditions, the ownership- and the agency-statements were compared against zero by means of the Wilcoxon signed-rank test in each group. Moreover, to investigate the relation between ownership and agency within the conditions, agency-statements were compared with the ownership-statements in each condition and in each group separately by means of the Wilcoxon signed-rank test.

Second, the Friedman test was used to analyze the factor condition (Active Synchronous Congruent, Passive Synchronous Congruent, Active Synchronous Incongruent, and Active Asynchronous Congruent) separately for the ownership-statements and the agency-statements in each group. *Post hoc* comparisons were performed using the Wilcoxon signed-rank test. This analysis allowed to test whether the sense of body ownership and the sense of agency varied across experimental conditions.

Third, the Mann–Whitney *U* test was used to compare the ownership-statements, ownership-controls, agency-statements and agency-controls between the two groups (FMD patients and healthy controls) across the four conditions.

Finally, the Spearman correlation was performed in order to investigate whether ownership-statements and agency-statements scores were related to disease duration in FMD patients.

Bonferroni correction for multiple comparisons was applied where necessary. All tests were two-tailed. *P*-values ≤ 0.05 were considered statistically significant. Additional analyses are reported in the Supplementary Materials.

## Results

Preliminary analyses showed that the two groups were comparable for age [*t*(40) = 1.511; *p* = 0.139] and gender (χ^2^ = 0.778; *p* = 0.378) distribution.

### Reliability of the mRHI Paradigm

#### FMD Group

**FIGURE 2:**
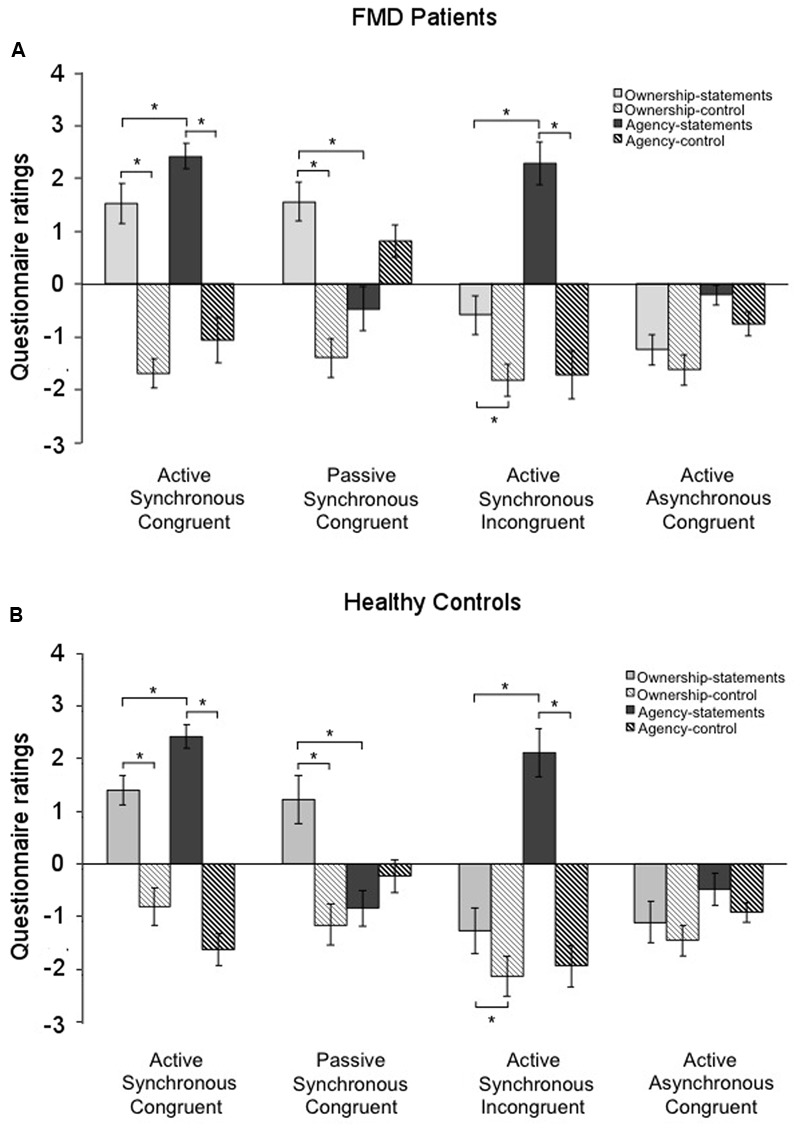
Questionnaire ratings in the two groups. Mean scores for the ownership-statements (light gray columns), ownership-controls (light gray striped columns), agency-statements (dark gray columns), and agency-controls (dark gray striped columns) in the functional movement disorders group **(A)** and the healthy controls group **(B)**. Gray lines and asterisks show significant comparisons within conditions. Note that in both groups the Passive Synchronous Congruent condition elicited ownership but not agency, and that conversely the Active Synchronous Incongruent condition elicited agency but not ownership, suggesting a double dissociation between agency and ownership. Error bars indicate standard errors.

In the FMD group, ownership-statements were rated significantly higher than ownership-control in the Active Synchronous Congruent condition (ownership-statements, 1.52 ± 0.39; ownership-control, -1.68 ± 0.28; *p* < 0.001) and in the Passive Synchronous Congruent condition (ownership-statements, 1.56 ± 0.37; ownership-control, -1.39 ± 0.31; *p* = 0.001). Interestingly, ownership-statements were rated significantly higher than ownership-controls also in the Active Synchronous Incongruent condition (ownership-statements, -0.58 ± 0.42; ownership-control, -1.81 ± 0.29; *p* < 0.017), which usually evokes agency but not ownership. In this case, however, the mean score of ownership-statements was negative, indicating a general disagreement with the statements assessing ownership (**Figure 2A**). Agency-statements were rated significantly higher than agency-control in the Active Synchronous Congruent (agency-statements, 2.42 ± 0.24; agency-control, -1.05 ± 0.43; *p* < 0.001) and in the Active Synchronous Incongruent conditions (agency-statements, 2.28 ± 0.19; agency-control, -1.71 ± 0.22; *p* < 0.001) (**Figure 2A**).

#### Healthy Control Group

Similar results were found in the control group, with ownership-statements having higher scores than ownership-control in the Active Synchronous Congruent condition (ownership-statements, 1.40 ± 0.28; ownership-control, -0.81 ± 0.28; *p* < 0.001), in the Passive Synchronous Congruent condition (ownership-statements, 1.21 ± 0.43; ownership-control, -1.15 ± 0.38; *p* < 0.001) and in the Active Synchronous Incongruent condition (ownership-statements, -1.27 ± 0.38; ownership-control, -2.13 ± 0.29; *p* = 0.002) (**Figure 2B**). Similarly, agency-statements were rated significantly higher than agency-control in the Active Synchronous Congruent (agency-statements, 2.42 ± 0.22; agency-control, -1.63 ± 0.30; *p* < 0.001) and in the Active Synchronous Incongruent conditions (agency-statements, 2.11 ± 0.30; agency-control, -1.94 ± 0.29; *p* < 0.001) (**Figure 2B**). These results show that the paradigm was suitable to successfully elicit both ownership and agency in the two groups.

Additional statistical details are reported in **Table 3**.

**Table 3:** Wilcoxon signed-rank test comparisons between ownership and agency statements and their respective control statements in each condition for both groups.

	Active Synchronous	Passive Synchronous	Active Synchronous	Active Asynchronous
	Congruent	Congruent	Incongruent	Congruent
**FMD group**				
Ownership statements vs. Ownership controls	Z = -3.757; *p* < 0.001	Z = -3.427; *p* = 0.001	Z = -2.379; *p* = 0.017	Z = -0.880; *p* = 0.379
Agency statements Vs. Agency controls	Z = -3.542; *p* < 0.001	Z = -1.700; *p* = 0.089	Z = -4.019; *p* < 0.001	Z = -0.916; *p* = 0.360
Ownership statements vs. Agency statements	Z = -2.534; *p* = 0.011	Z = -3.082; *p* = 0.002	Z = -3.827; *p* < 0.001	Z = -2.508; *p* = 0.012
**HC group**				
Ownership statements vs. Ownership controls	Z = -3.666; *p* < 0.001	Z = -3.626; *p* < 0.001	Z = -3.163; *p* = 0.002	Z = -0.427; *p* = 0.669
Agency statements vs. Agency controls	Z = -3.925; *p* < 0.001	Z = -0.987; *p* = 0.324	Z = -3.927; *p* < 0.001	Z = -1.442; *p* = 0.149
Ownership statements vs. Agency statements	Z = -3.341; *p* = 0.001	Z = -2.834; *p* = 0.005	Z = -3.788; *p* < 0.001	Z = -1.661; *p* = 0.097

### Agency and Ownership within Each Condition

#### FMD Group

Wilcoxon signed rank test used to test whether the scores at the experimental statements were significantly different from zero, revealed that agency-statements differed from zero in the Active Synchronous Congruent (*p* < 0.001) and in the Active Synchronous Incongruent conditions (*p* < 0.001). Moreover, scores at the ownership-statements were significantly different from zero in the Active Synchronous Congruent (*p* = 0.004), Passive Synchronous Congruent (*p* = 0.001) and Active Asynchronous Congruent (*p* = 0.006) conditions. Interestingly, ownership-statements did not differ from zero in the Active Synchronous Incongruent condition (*p* = 0.177), thus suggesting uncertainty about ownership over the rubber hand.

The comparison between agency- and ownership-statements within each condition revealed that the agency-statements scores for the FMD group were higher than the ownership-statements scores in the Active Synchronous Congruent (*p* = 0.011), Active Asynchronous Congruent (*p* = 0.012), and Active Synchronous Incongruent conditions (*p* < 0.001). Conversely, in the Passive Synchronous Congruent condition, the ownership-statements scores were higher than the agency-statements scores (*p* = 0.002) (**Figure 2A**).

#### Healthy Control Group

By comparing the experimental statements against zero, we found that agency-statements scores significantly differed from zero in the Active Synchronous Congruent (*p* < 0.001) and in the Active Synchronous Incongruent conditions (*p* < 0.001). The ownership-statements scores differed from zero in the Active Synchronous Congruent (*p* = 0.001), Passive Synchronous Congruent (*p* = 0.023), Active Asynchronous Congruent (*p* = 0.024) and Active Synchronous Incongruent (*p* = 0.009) conditions.

By comparing the ownership- and agency-statements scores, we found that agency-statements scores were higher than the ownership-statements scores in the Active Synchronous Congruent (*p* = 0.001) and in the Active Synchronous Incongruent conditions (*p* < 0.001), but not in the Active Asynchronous Congruent condition (*p* = 0.097) (**Figure 2B**). Again, in the Passive Synchronous Congruent condition, the ownership-statements scores were higher than the agency-statements scores (*p* = 0.005) (**Figure 2B**). These results show a double dissociation between agency and ownership, since in the Passive Synchronous Congruent condition we could elicit ownership but not agency; conversely, in the Active Synchronous Incongruent condition we could elicit agency but not ownership.

See **Table 3** for additional statistical details.

### Agency and Ownership across Conditions

#### FMD Group

**FIGURE 3:**
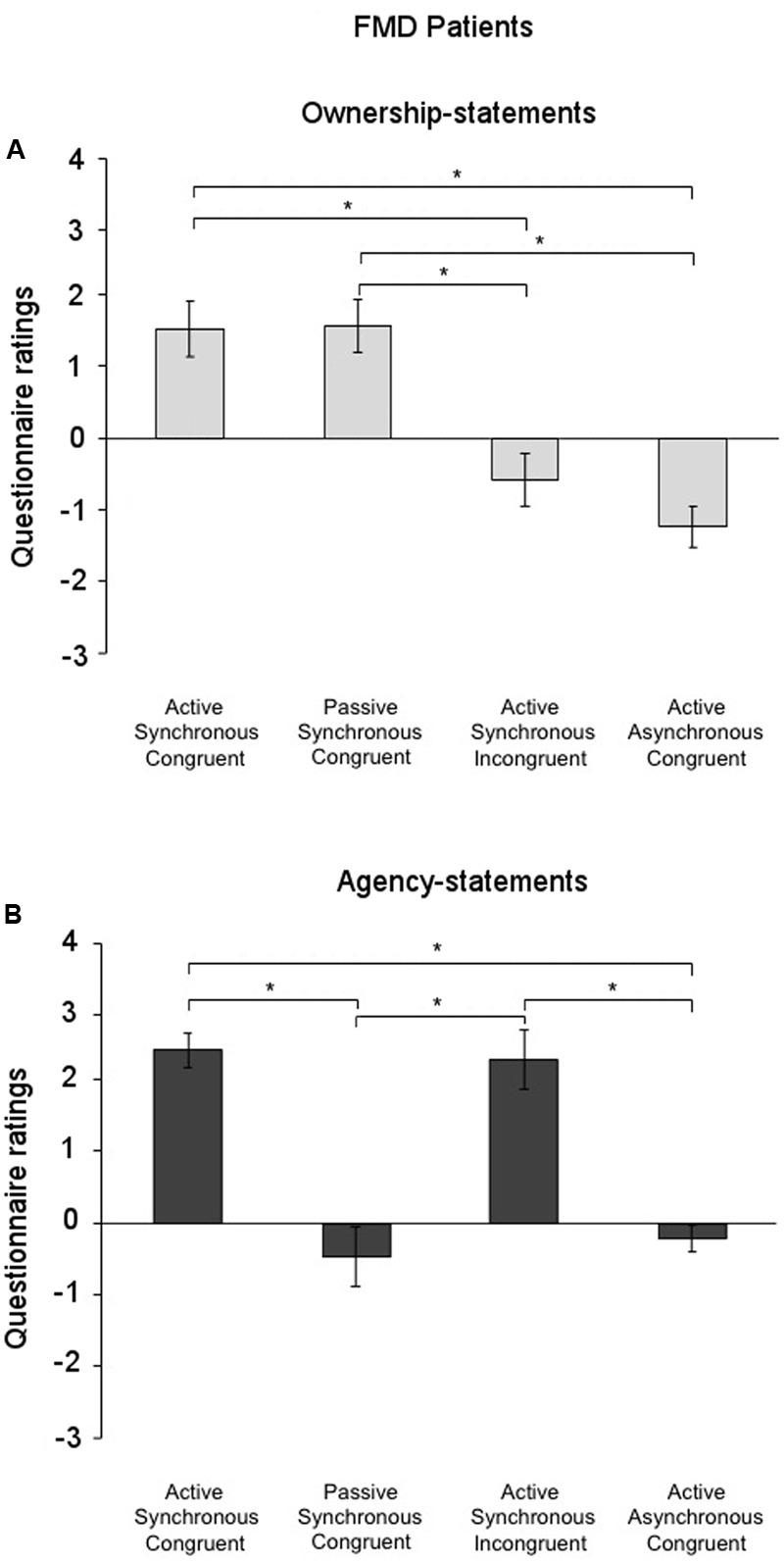
Ownership and agency across conditions in the functional movement disorders group. **(A)** Mean scores for the ownership-statements and **(B)** the agency-statements across conditions in the functional movement disorders group. Gray lines and asterisks show significant comparisons across conditions. The results showed that agency and ownership were differently elicited by each condition. Error bars indicate standard errors.

The Friedman test to analyze the factor condition separately for the ownership-statements and the agency-statements, revealed that in the FMD group the factor condition was significant for both the ownership- [χ^2^(3) = 29.09; *p* < 0.001] and the agency-statements [χ^2^(3) = 36.96; *p* < 0.001]. *Post hoc* pairwise comparisons (critical *p* ≤ 0.016 after Bonferroni correction) showed that the ownership-statement scores in both the Active Synchronous Congruent (1.52 ± 0.39) and the Passive Synchronous Congruent conditions (1.56 ± 0.37) were higher than in the Active Asynchronous Congruent (-1.24 ± 0.37) and the Active Synchronous Incongruent conditions (-0.58 ± 0.42) (all comparisons, *p* < 0.002) (**Figure 3A**). Furthermore, the agency-statement scores were higher in the Active Synchronous Congruent (2.42 ± 0.24) and the Active Synchronous Incongruent conditions (2.28 ± 0.19) than in the Active Asynchronous Congruent (-0.20 ± 0.41) and the Passive Synchronous Congruent conditions (-0.46 ± 0.41) (all comparisons, *p* < 0.001) (**Figure 3B**).

#### Healthy Control Group

**FIGURE 4:**
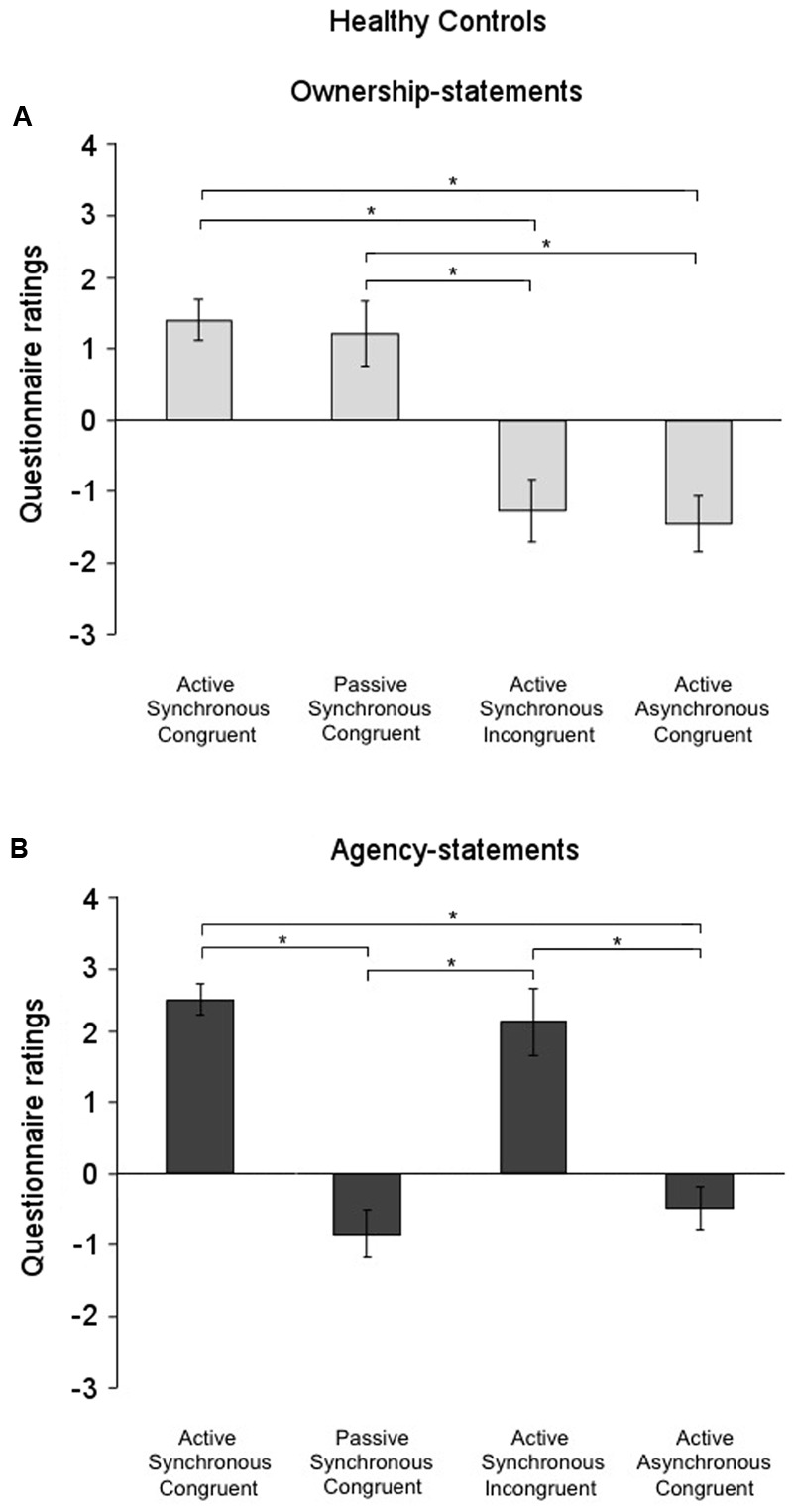
Ownership and agency across conditions in the healthy controls group. **(A)** Mean scores for the ownership-statements and **(B)** the agency-statements across conditions in the healthy controls group. Gray lines and asterisks show significant comparisons across conditions. In the functional movement disorders group, agency and ownership were differently elicited by each condition. Error bars indicate standard errors.

The factor condition was significant for both the ownership- [χ^2^(3) = 32.72; *p* = 0.000] and the agency-statements [χ^2^(3) = 33.46; *p* < 0.001] also in the control group. As found for the FMD group, *post hoc* comparisons (critical *p* ≤ 0.016 after Bonferroni correction) showed that the ownership-statement scores were higher in both the Active Synchronous Congruent (1.40 ± 0.28) and the Passive Synchronous Congruent conditions (1.21 ± 0.43) than in the Active Asynchronous Congruent (-1.11 ± 0.34) and the Active Synchronous Incongruent conditions (-1.27 ± 0.38) (all comparisons, *p* < 0.001) (**Figure 4A**). Agency-statement scores were higher in the Active Synchronous Congruent (2.42 ± 0.22) and the Active Synchronous Incongruent (2.11 ± 0.30) conditions than in the Active Asynchronous Congruent (-0.49 ± 0.34) and the Passive Synchronous Congruent conditions (-0.84 ± 0.46) (all comparisons, *p* < 0.001) (**Figure 4B**).

These results show that agency and ownership were differently elicited in specific experimental conditions.

See **Table 4** for additional statistical details on the *post hoc* comparisons.

**Table 4:** Wilcoxon signed-rank comparisons between conditions for ownership and agency statements in each group.

	Passive Synchronous Congruent	Active Synchronous Incongruent	Active Asynchronous Congruent
**FMD group**			
**Ownership-statements**			
Active Synchronous Congruent	Z = -0.314; *p* = 0.753	Z = -3.139; *p* = 0.002	Z = -3.705; *p* < 0.001
Passive Synchronous Congruent	-	Z = -3.556; *p* < 0.001	Z = -3.642; *p* < 0.001
Active Synchronous Incongruent	-	-	Z = -1.410; *p* = 0.161
**Agency-statements**			
Active Synchronous Congruent	Z = -3.729; *p* < 0.001	Z = -0.509; *p* = 0.615	Z = -3.810; *p* < 0.001
Passive Synchronous Congruent	-	Z = -3.689; *p* < 0.001	Z = -0.605; *p* = 0.545
Active Synchronous Incongruent	-	-	Z = -3.422; *p* = 0.001
**HC group**			
**Ownership-statements**			
Active Synchronous Congruent	Z = -0.349; *p* = 0.727	Z = -3.828; *p* < 0.001	Z = -3.826; *p* < 0.001
Passive Synchronous Congruent	-	Z = -3.642; *p* < 0.001	Z = -3.282; *p* = 0.001
Active Synchronous Incongruent	-	-	Z = -0.103; *p* = 0.918
**Agency-statements**			
Active Synchronous Congruent	Z = -3.470; *p* = 0.001	Z = -1.046; *p* = 0.294	Z = -3.964; *p* < 0.001
Passive Synchronous Congruent	-	Z = -3.388; *p* = 0.001	Z = -0.676; *p* = 0.499
Active Synchronous Incongruent	-	-	Z = -3.885; *p* < 0.001

### Agency and Ownership between Groups

**FIGURE 5:**
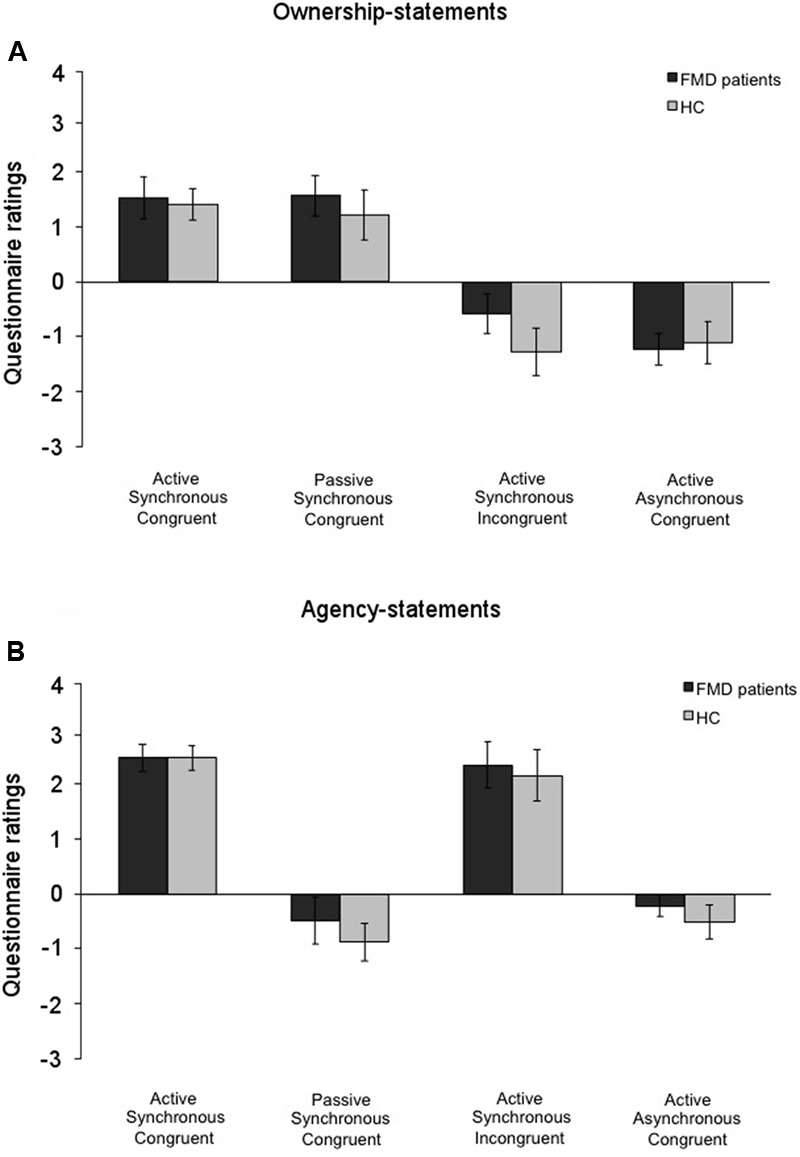
Ownership and agency for all the conditions in the functional movement disorders and the healthy controls groups. Mean scores for the**(A)** ownership-statements and **(B)** the agency-statements in the functional movement disorders group (FMD; black columns) and the healthy controls group (HC; gray columns). The two groups showed similar patterns of response. Error bars represent standard errors.

The Mann–Whitney *U* test for comparisons between the two groups (FMD and HC) revealed similar scores for ownership in the Active Synchronous Congruent (FMD: 1.52 ± 0.39; HC: 1.40 ± 0.28; *p* = 0.423), the Passive Synchronous Congruent (FMD: 1.56 ± 0.37; HC: 1.21 ± 0.43; *p* = 0.626), the Active Synchronous Incongruent (FMD: -0.58 ± 0.42; HC: -1.27 ± 0.38; *p* = 0.207), and the Active Asynchronous Congruent conditions (FMD: -1.24 ± 0.37; HC: -1.11 ± 0.34; *p* = 0.939) (**Figure 5A**). The two groups had also similar scores with regard to agency in the Active Synchronous Congruent (FMD: 2.42 ± 0.24; HC: 2.42 ± 0.22; *p* = 0.697), the Passive Synchronous Congruent (FMD: -0.46 ± 0.41; HC: -0.84 ± 0.46; *p* = 0.362), the Active Synchronous Incongruent (FMD: 2.28 ± 0.19; HC: 2.11 ± 0.30; *p* = 0.895), and the Active Asynchronous Congruent condition (FMD: -0.20 ± 0.41; HC: -0.49 ± 0.34; *p* = 0.696) (**Figure 5B** and Supplementary Figure 1). These results indicate that the FMD patients rated their subjective feeling of agency and ownership similar to the controls. Moreover, similar results were found for the control statements as well, which were rated similarly by both groups (all, *p* > 0.065), suggesting that, like the HC group, the FMD patients also experienced other perceptual effects related to the mRHI paradigm.

### Correlation between Disease Duration, Agency- and Ownership-Statements in FMD Patients

Ownership-statements did not correlate with disease duration in any conditions (Active Synchronous Congruent: *r* = 0.378, *n* = 20, *p* = 0.100; Passive Synchronous Congruent: *r* = 0.171, *n* = 20, *p* = 0.472; Active Synchronous Incongruent: *r* = 0.066, *n* = 20, *p* = 0.781; Active Asynchronous Congruent: *r* = 0.180, *n* = 20, *p* = 0.448). Similarly, we did not found significant correlations between agency-statements and disease duration (Active Synchronous Congruent: *r* = 0.065, *n* = 20, *p* = 0.785; Passive Synchronous Congruent: *r* = 0.345, *n* = 20, *p* = 0.136; Active Synchronous Incongruent: *r* = 0.010, *n* = 20, *p* = 0.966; Active Asynchronous Congruent: *r* = 0.230, *n* = 20, *p* = 0.329).

## Discussion

To our knowledge, this is the first study to investigate the sense of agency over bodily movements, the sense of body ownership, and their interaction in FMD patients by means of the mRHI. The data from the healthy controls confirmed the reliability of the mRHI paradigm in measuring sense of agency and sense of body ownership: the agency-statements and the ownership-statements significantly differed from their respective control statements in the Active Synchronous Congruent, Active Synchronous Incongruent, and Passive Synchronous Congruent conditions. We also found that all participants experienced a strong sense of agency and illusion of body ownership in the Active Synchronous Congruent condition, whereas in the Active Asynchronous Congruent condition, they generally disagreed with both the agency- and the ownership-statements, indicating that adding a temporal delay between performed and observed movement disrupted the sense of both agency and ownership. Furthermore, a dissociated pattern of results was observed in the Passive Synchronous Congruent and the Active Synchronous Incongruent conditions. In the Passive Synchronous Congruent condition, participants experienced an illusory sense of body ownership but not agency. Conversely, in the Active Synchronous Incongruent condition, they perceived a strong sense of agency but not a sense of body ownership. These results are in line with previous mRHI studies (Kalckert and Ehrsson, 2012, 2014) and indicate a double dissociation between ownership and agency, with the former not necessarily implying the latter and vice versa.

The most interesting finding was the lack of differences between the two groups with regard to sense of agency. This finding was unexpected, especially in the experimental conditions involving active movements. Previous studies demonstrated that execution of active movements requires matching processes of predicted and actual sensory feedback (Blakemore et al., 1998; Frith et al., 2000; van den Bos and Jeannerod, 2002; Farrer et al., 2008; Chambon et al., 2014), which are known to be altered in FMD patients (Voon et al., 2010, 2011; Edwards et al., 2013; Maurer et al., 2016).

In our paradigm, we expected a reduced sense of agency in the FMD patients specifically in the Active Synchronous Congruent and in the Active Synchronous Incongruent conditions in which these processes should occur.

The lack of difference between the two groups could be explained by the nature of the paradigm applied. In the mRHI used here, the sense of agency was evaluated with an explicit measure, whereas previous studies used implicit paradigms (Edwards et al., 2011; Kranick et al., 2013; Pareés et al., 2014; Macerollo et al., 2015). As a general consideration, the use of implicit paradigms offers several advantages, for instance, the possibility to target and measure specific correlates of voluntary action (e.g., sensory attenuation) without requiring participants to explicitly focus on agency (Wolpe and Rowe, 2014; Haggard, 2017). This allows to potentially exclude confounding factors, such as cognitive biases (e.g., tendency to overestimate one’s own agency) or contextual cues, which could influence explicit judgment of agency (Haggard, 2017). On the other hand, implicit measures do not allow to assess the subjective experience of control which is, instead, captured by explicit measures (Moore et al., 2012; Dewey and Knoblich, 2014). With regard to the clinical population in our study (i.e., FMD patients), explicit measures allow to capture what happens in clinical practice, when FMD patients explicitly report a lack of control over their motor symptoms. Moreover, by using explicit measures we were able to specifically assess an aspect of the sense of agency which implicit measures, by nature, are unable to detect (e.g., the subjective experience of control over bodily movements). Nonetheless, one could argue that since our findings are based on explicit measures, they could have been biased by the participants’ compliance and suggestibility. However, the difference in scores between the ownership- and agency-statements and their respective control statements suggests that the participants answered truthfully and not because of mere compliance or suggestibility. Explicit and implicit measures of agency have been found to dissociate and imply different underlying mechanisms (Dewey and Carr, 2013). More precisely, the implicit measures of agency (e.g., sensory attenuation and intentional binding) are thought to capture the so-called “feeling of agency” (i.e., non-conceptual feeling of being the agent of a certain action) that relies on low-level sensorimotor processes involving efferent motor cues and sensory feedback (Synofzik et al., 2008; Moore et al., 2012). However, the sense of agency does not involve only a non-conceptual level (i.e., “feeling of agency”), but also a conceptual level, that is the “judgment of agency” (Synofzik et al., 2008; Moore et al., 2012). The explicit measures of agency reflect this “judgment of agency” (i.e., the explicit attribution of agency) that involves higher-order sources of information like beliefs and contextual cues (Synofzik et al., 2008; Desantis et al., 2011, 2012; Moore et al., 2012). Explicit and implicit aspects of agency also differ with regards to the brain areas that they involve. More precisely, while the pre-supplementary motor area within a frontal-striatal circuit is involved in implicit aspects of agency (Wolpe et al., 2016), the insula and parietal lobe are involved in the explicit aspects of agency (Farrer and Frith, 2002; Farrer et al., 2003).

Hence, implicit and explicit measures of agency allow to capture different aspects.

Our findings suggest that the explicit component of sense of agency is preserved. Why this is so is still unclear. One hypothesis is related to the potential role of other cognitive factors, like prior beliefs, in differentially influencing the explicit sense of agency for normal voluntary movements and for motor symptoms. In this regard, it was suggested that a patient’s feeling of control over motor symptoms is undermined by the belief that the symptom has an organic basis (Kranick and Hallett, 2013). We speculate that this prior belief could affect the explicit judgment of agency for motor symptoms, but not for normal voluntary movements like those required in our task (e.g., finger tapping).

Our findings also contrast with previous studies that targeted other clinical populations (e.g., schizophrenia), which present with abnormalities in the sense of agency (Franck et al., 2001; Thakkar et al., 2011). For instance, Franck et al. (2001) found that schizophrenic patients made more self-attribution errors than healthy controls on an action recognition task, demonstrating that schizophrenic patients have difficulty in distinguishing their own action from others’ actions. Such is not the case in FMD patients. Indeed, as demonstrated for the first time by our study, patients with FMD are able to distinguish a self-generated bodily action (i.e., Active Synchronous Congruent condition, Active Synchronous Incongruent condition) from an externally induced action (i.e., Passive Synchronous Congruent condition).

We also found that the two groups were comparable for the sense of body ownership, suggesting that this cognitive function is preserved in FMD patients. This observation is shared by another recent study that used the classical RHI paradigm (Demartini et al., 2016). In the RHI paradigm, the illusory sense of body ownership over the artificial hand is induced by stroking a static visible rubber hand and the participant’s hidden hand simultaneously (Botvinick and Cohen, 1998). In this context, the multisensory integration of visual, proprioceptive, and tactile information grounds the illusion. Demartini et al. (2016) found a similar amount of illusion in FMD patients and healthy controls, suggesting that the sense of body ownership is efficient in FMD. With our mRHI study, we add new insight along this line by demonstrating that the sense of body ownership is preserved in FMD even when visual and proprioceptive sensory information interact with the motor system (rather than with tactile information).

Moreover, previous studies used the RHI to investigate interaction between the motor system and the sense of body ownership in other movement disorders (e.g., hemiplegia) (Lenggenhager et al., 2012; Scandola et al., 2014; Tidoni et al., 2014; Burin et al., 2015). Interestingly, these studies demonstrated that despite abnormalities in the motor system, patients subjectively experienced a strong illusion (Lenggenhager et al., 2012; Scandola et al., 2014; Tidoni et al., 2014; Burin et al., 2015). Caution is warranted when comparing our study with previous works targeting movement disorders due to an organic disease (e.g., hemiplegia). Our findings of preserved illusory body ownership in FMD add further insights into the relationship between the presence of motor symptoms and the sense of body ownership. Namely, in line with a previous study (Demartini et al., 2016) no alteration of this cognitive function has been found in FMD.

Although the main analyses did not show significant differences in agency-statements and ownership-statements between the FMD and HC groups, additional analysis revealed a different pattern of responses between the two groups with regard to one specific condition: the Active Synchronous Incongruent. Of note, in this condition, the rubber hand is placed in an anatomically implausible posture (180° rotated with respect to the participant’s body), thus usually preventing the illusory embodiment of the rubber hand. In this condition, however, participants can actively move the rubber hand, thus favoring a sense of agency over the observed hand. As expected, the scores for the HC group on the ownership-statements were different from 0 in a negative direction, suggesting that they denied a sense of body ownership over the rubber hand. Conversely, the FMD group scores were not different from 0, revealing uncertainty about ownership over the rubber hand. This pattern of results may suggest that in FMD patients the execution of an active movement makes the processing of an incompatible rubber hand less clear-cut, thus creating uncertainty about a possible illusory sense of ownership. A possible interpretation of this finding could be related to decision-making processes. More precisely, a previous study using an abstract probabilistic reasoning task showed that FMD patients make decisions on the basis of less evidence compared to healthy controls (Pareés et al., 2012). It was suggested that this style of reasoning, called “jumping to conclusion” bias, derives from the overestimation of the sensory data, which causes an inappropriate updating of internal models (Pareés et al., 2012). In the context of our study, we speculate that in the Active Synchronous Incongruent condition, the sensory information coming from the moving body part, together with the similarities between the rubber hand and the own hand (e.g., similar shape), were taken as sufficient evidence to jump to the conclusion that the rubber hand could potentially be the own hand. Why this is the case only in the Active Synchronous Incongruent condition is not clear and deserves further investigation.

This study has several limitations. First, since we used explicit (i.e., questionnaire) rather than implicit measures (e.g., proprioceptive drift, sensory attenuation, intentional biding) of agency and ownership, we cannot directly compare explicit and implicit RHI effects in our sample, which limits the generalizability of our results. Future mRHI studies in FMD using also implicit measures of both functions would add further information about the mechanisms underlying self-attribution of bodily action in FMD. Second, the heterogeneous sample of patients (10 with functional tremor alone or associated with other functional symptoms, 11 with gait disorders) limits detailed evaluation of potential differences in sense of agency in relation to the type and localization of functional motor symptoms, especially in our study where we tested the sense of agency for upper limb movements. It should be noted, however, that our sample was similar to those of previous studies in which alterations in the implicit sense of agency were found (Kranick et al., 2013). Third, the participants rated their subjective feelings on a 7-point Likert scale. Although, the scale was able to detect differences between groups (e.g., schizophrenic patients vs. healthy controls) in a previous RHI study (Thakkar et al., 2011), it could be argued that the scale was not sensitive enough to capture potential alterations in the FMD patients in the present study. Fourth, our task was developed from previous studies in which one trial per condition was tested. Since the patient sample was characterized by between- as well as within-subject variability in performance, having more than one trial per condition could have helped to reduce variability and obtain more consistent results. By the same token, adding more repetitions of the same trial would have increased the duration of the overall experiment, with the potential risk of attenuating the patients’ compliance to continue with the session due to mental fatigue and diminished attentional resources. Fifth, there were only two men in our sample of 21 patients, and this may limit the generalizability of our findings to male FMD patients. This feature (i.e., unbalanced gender distribution) is shared with the majority of previous studies (e.g., Edwards et al., 2013; Pareés et al., 2014; Macerollo et al., 2015), and might reflect the observation that FMDs are significantly more common among women than men (Peckham and Hallet, 2009). Sixth, the lack of significant difference between patients and controls in our study was derived by applying a conventional statistical approach for data analysis. Previous studies in which a similar paradigm was applied (Kalckert and Ehrsson, 2012, 2014; Louzolo et al., 2015) used the same statistical approach. Hence, we thought that it was suitable also to our explorative study, as it gave us the possibility to interpret our findings based on the previous literature. We acknowledge, however, that this does not allow to state that the null hypothesis is true and therefore that the two groups are the same. Other statistical approaches, like the Bayes factor analysis, could help in the future to disambiguate how much likely the null hypothesis is with respect to the hypothesis that the groups differ.

These limitations notwithstanding, this is the first empirical study to suggest that the subjective experience of agency for normal voluntary movements is preserved in FMD, as assessed by the moving Rubber Hand Illusion paradigm. Together with previous work, our study depicts the sense of agency as a complex cognitive function in which implicit and explicit aspects can differentially influence the subjective experience of motor control in FMD patients. Two forms of dissociation may be distinguished: a dissociation between the implicit and the explicit sense of agency for normal voluntary movements in FMD and a dissociation between the explicit sense of agency for normal voluntary movements and for functional motor symptoms. Further studies are needed to elucidate the interactions between the implicit and the explicit components of the sense of agency in FMD and also to reveal the mechanisms underlying a possible dissociation in the explicit sense of agency between normal voluntary movement and functional motor symptoms.

## Author Contributions

MF, MZ, and MT: conception, design and organization of the research project. AM, FB, CD, and FS: organization of the research project. AM, FB, and MF: organization and acquisition of the data. AM, MF, and MZ: design and execution of data analysis, and interpretation of the data. AM and MF: writing the first draft and revision of it based on other authors’ critique. MT, MZ, CD, and FS: revising the work for critique, and agreement to be accountable for all aspects of the work in ensuring that questions related to the accuracy and integrity of any part of the work were appropriately investigated. AM, FB, CD, FS, MF, and MT: final approval of the version to be published.

## Conflict of Interest Statement

The authors declare that the research was conducted in the absence of any commercial or financial relationships that could be construed as a potential conflict of interest.
