# Supplementary material for: The Moving Rubber Hand Illusion Reveals that Explicit Sense of Agency for Tapping Movements Is Preserved in Functional Movement Disorders
Source: Front Hum Neurosci. 2017 Jun 6;11:291. doi: 10.3389/fnhum.2017.00291 (PMC5459911; doi:10.3389/fnhum.2017.00291)
Supplement: Supplementary file 1 [file Data_Sheet_1.DOCX]

Supplementary Material

The Moving Rubber Hand Illusion Reveals that Explicit Sense of Agency for tapping movements is Preserved in Functional Movement Disorders

**Angela Marotta*, Federica Bombieri, Massimiliano Zampini, Federico Schena, Carlo Dallocchio, Mirta Fiorio^§^, Michele Tinazzi^§^**

^§^These authors equally contributed to this work.

**Correspondence:** Angela Marotta: angela.marotta@univr.it

**Supplementary data analysis**

In order to test whether the scores at the agency-statements were different depending on specific symptoms presented by FMD patients, we defined two sub-groups of patients, characterized by similar symptoms. In this way, we identified one sub-group of patients (n=10; 9 women; mean age ± standard deviation, 36.10 ± 14.48 years) presenting functional tremor, alone or associated with other functional motor symptoms (e.g., gait disorder, dystonia) (FMD with tremor), and one sub-group of patients (n=11; 10 women; 42.55 ± 10.91 years), who did not present tremor (FMD without tremor). We then used the Kruskall-Wallis test to explore potential differences between the two sub-groups of patients and healthy controls with regard to agency-statements. The Mann-Whitney U test was used for post-hoc comparisons between groups in case of significant group effects. Bonferroni correction for multiple comparisons was applied where necessary. All tests were two-tailed. P values ≤ 0.05 were considered statistically significant.

**Supplementary results**

Kruskall-Wallis test used for comparing agency-statements between the two-subgroups of FMD patients (i.e., FMD-tremor, FMD-no tremor) and the HC participants was not significant in any condition (Active Synchronous Congruent: χ^2^ = 0.247; *p* = 0.884; Passive Synchronous Congruent: χ^2^ = 1.487, *p* = 0.475; Active Synchronous Incongruent: χ^2^ = 0.477, *p* = 0.788; Active Asynchronous Congruent: χ^2^ = 0.154, *p* = 0.926).

These analyses revealed no differences between different sub-groups of patients, nor between each FMD sub-groups (i.e., FMD with tremor, FMD without tremor) and healthy controls. Hence, it appears that having functional tremor or gait disorder did not influence the subjective experience of agency reported by our FMD patients.

**Supplementary figure**

**
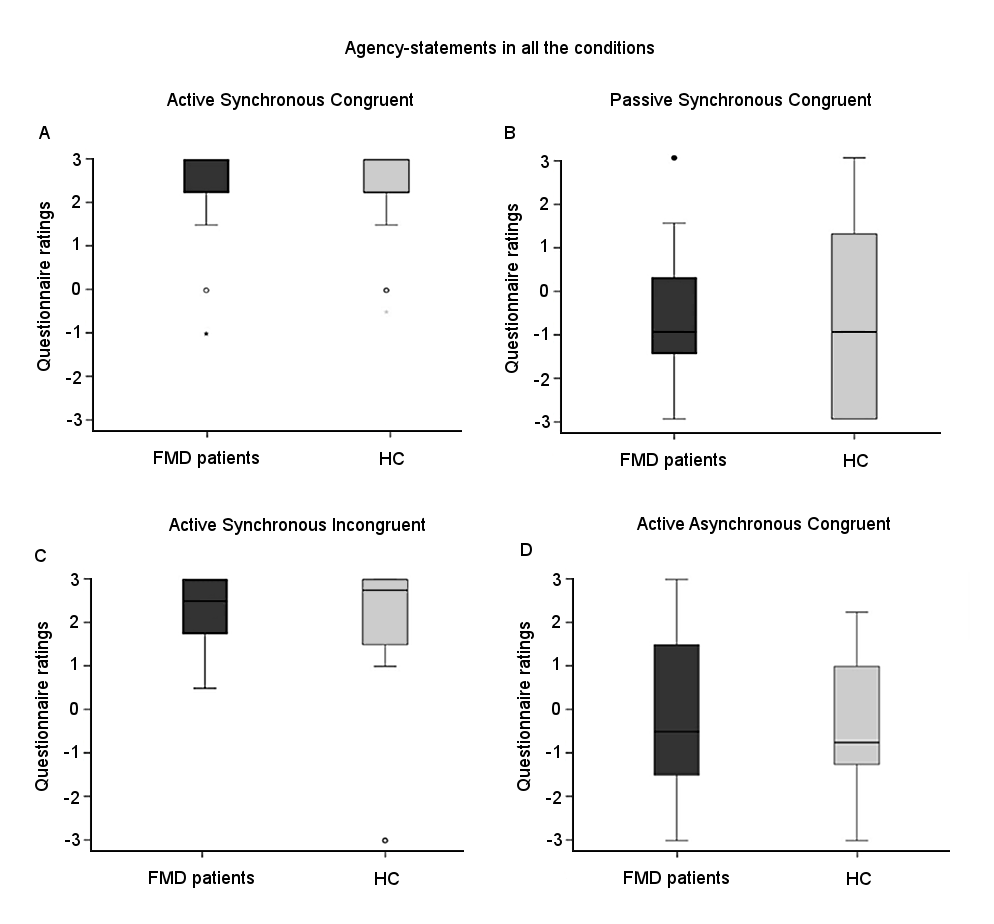
**

**Supplementary Figure 1.** Agency-statements ratings in the FMD and HC groups for the A) Active Synchronous Congruent, B) Passive Synchronous Congruent, C) Active Synchronous Incongruent and D) Active Asynchronous Congruent. Of note, the within-group variability median value of agency-statements was similar between FMD patients and healthy controls. Points represent outliers (e.g., values that fall between 1.5 and 3 times above or below the interquartile range). Asterisks represent extreme outliers (e.g., values that are more than three times above or below the interquartile range).
